# Mining of candidate genes related to prolificacy in Jining grey goats using transcriptomics

**DOI:** 10.1186/s12864-025-12284-4

**Published:** 2025-12-15

**Authors:** Jingyi Mao, Junmin He, Yifan Ren, Xue Li, Cunming Yang, Guifen Liu, Guoping Zhang, Chen Wei, Wenhao Zhang, Ming Wang, Fumei Nie, Kechuan Tian, Xixia Huang

**Affiliations:** 1 Institute of Animal Husbandry and Veterinary Medicine, Shandong Key Laboratory of Livestock and Poultry Breeding, Shandong Academy of Agricultural Sciences, Jinan, 250100 China; 2 College of Animal Science, Xinjiang Agricultural University, Urumqi, 830052 China; 3 College of Life Sciences, Xinjiang Normal University, Urumqi, 830017 China

**Keywords:** Jining grey goat, Multiple birth traits, RNA-seq, Gene screening, Meta-analysis

## Abstract

**Background:**

The Jining grey goat is a renowned local goat breed in China that has exceptional reproductive performance; thereby, it serves as an ideal animal model for studying prolificacy in goats. Recent research on the reproductive traits of Jining grey goats has focused primarily on variations in the expression of key genes, hormone regulatory mechanisms, and breeding techniques, with the aim of enhancing reproductive performance through molecular marker-assisted breeding.

**Methods:**

In this study, 8 healthy adult female Jining grey goats of similar age and weight were selected. Ovarian and uterine tissues were collected from Jining grey goats in the high litter size group (HL, high size ≥ 3 in three consecutive litters, *n* = 4) and the low litter size group (LL, litter size = 1 in three consecutive litters, *n* = 4) for transcriptome sequencing. Bioinformatics analysis was conducted to investigate key regulatory pathways and candidate genes in ovarian and uterine tissues. Ultimately, 5 pathways (cAMP signalling pathway, neuroactive ligand‒receptor interaction, ABC transporters, cytokine‒cytokine receptor interaction and metabolic pathways) and 10 candidate genes (*3BHSD*, *CLSTN2*, *BMP5*, *DRD1*, *CXCL14*, *CFAP43*, *WNT10B*, *ENO4*, *GABRA1* and the *DNAH1* gene) were revealed to be associated with multiple births in Jining grey goats. RT‒qPCR was used to verify the sequencing results, and the relative expression levels of the 10 candidate genes in different tissues were compared. Among them, *3BHSD*, *CLSTN2*, and *GABRA1* were found to be closely related to prolificacy in the Jining grey goat.

**Conclusion:**

In this study, key regulatory pathways and candidate genes influencing the prolificacy of Jining Grey goats were investigated through transcriptome analysis, providing in-depth insights into the molecular mechanisms through which the expression of genes related to prolificacy in different tissues affects reproductive performance. These findings provide a theoretical foundation and reference for the selective breeding of Jining grey goats.

**Supplementary Information:**

The online version contains supplementary material available at 10.1186/s12864-025-12284-4.

The Jining grey goat is a renowned local goat breed in Shandong Province, China. It is famous for its “four grey and one black” grey coat pattern, superior meat flavour, and exceptional reproductive performance [1], making it an ideal animal model for studying prolificacy in goats. Reproductive traits play a crucial role in the economic value of goats and directly impact the profitability of the goat industry [2]. Multiple birth traits is a reproductive trait that is a key breeding trait in goats. Factors influencing reproductive performance include litter size, conception rate, age, and health status, all of which are influenced by interactions between the environment and genetics and have complex regulatory mechanisms. Conventional breeding techniques often have disadvantages such as low efficiency, long cycles, and high difficulty, thus requiring the integration of molecular biological methods such as transcriptome sequencing (RNA-seq) and genome-wide association studies (GWASs) to explore key regulatory pathways and candidate genes related to reproductive traits in detail, thereby accelerating the molecular breeding of goats with high birth rates [3, 4]. The ovulation rate and litter size reflect the reproductive capacity of animals and have significant impacts on farm profitability and breeding program success [5]. The ovaries and uterus, important reproductive organs in goats, provide an internal environment conducive to embryo growth and development. The ovaries and uterus secrete key reproductive hormones, including oestrogen, oxytocin, progesterone, prolactin, and follicle-stimulating hormone [6–8]. Furthermore, these organs play crucial roles in regulating the mammalian endocrine system and the female oestrous cycle. Together, these functions collectively determine reproductive performance. A deeper understanding of the molecular mechanisms through which the ovaries and uterus can effectively improve the reproductive efficiency and economic benefits of goats is needed.

Currently, the molecular regulatory mechanisms that regulate fecundity in goats and the associated gene network interactions are not fully understood, which limits the success of genetic breeding programs. Therefore, the identification of genes and molecular markers associated with fertility in goats has become a research hotspot. Researchers have identified several candidate genes related to high prolificacy in sheep and goats, such as the *BMPRIB*, *GDF9*, *BMP15*, *ESR*, and *FSHR* genes [9–14]. In addition, research on goat reproduction has focused mainly on candidate genes expressed in the ovarian tissues of Jining grey goats, while the important role of the uterus in reproduction and the expression of genes between the ovary and uterus have not been sufficiently explored. Therefore, this study combines RNA-seq data from ovarian and uterine tissues with bioinformatics analysis to identify key candidate genes related to prolificacy in the Jining grey goat. The expression of these candidate genes may simultaneously influence the functions of both the ovary and uterus, thus exerting a dual impact on prolificacy in the Jining grey goat. In summary, RNA-seq and bioinformatics analyses of ovarian and uterine tissues were performed in this study to investigate the regulatory mechanisms of fecundity and the key candidate genes in Jining grey goats, providing a theoretical molecular foundation and reference markers for the development of new Jining grey goat lines with high prolificacy.

## Results

### Sequencing data quality control and reference genome comparison

The RNA from ovarian and uterine tissue samples of Jining grey goats was separately extracted and analysed, with concentrations and purities meeting the standards, ensuring quality compliance. cDNA libraries were subsequently constructed, and RNA-seq was performed on both ovarian and uterine tissues. The average data volumes for each sample were 11.22 G and 11.31 G, respectively, with raw read averages exceeding 11.24 million and 11.41 million, respectively. After quality control filtering, the clean read Q20 values were all above 95%, and the clean read Q30 values were all above 90%. The clean reads were then aligned to the *Capra hircus* reference genome, resulting in total alignment rates and unique alignment rates of over 91% and 86%, respectively. This preliminarily confirmed that the results were accurate and suitable for further experimental analysis.

### Correlation analyses

Correlation analysis was performed on the HL group (High litter size_Ovaries and High litter size_Uterus, HL_Os and HL_Us) and the LL group (Low litter size_Ovaries and Low litter size_Uterus, LL_Os and LL_Us) RNA-seq data, and the Pearson correlation coefficients between samples within the ovarian and uterine sample groups both exceeded 0.6, indicating that there was differences in the gene expression among samples from these two tissues. Additionally, the principal component analysis (PCA) based on the Bray-Curtis distance and PERMANOVA tests. The results revealed significant compositional differences between ovarian and uterine tissue groups (PERMANOVA: R² = 0.282 and 0.301, respectively; both *P* < 0.05). The PCA plot showed good intra-group homogeneity, indicating that samples within the same group were compositionally similar (Supplementary Fig. 1).

### Screening and analysis of candidate genes related to prolificacy in ovarian tissue

**Fig. 1:**
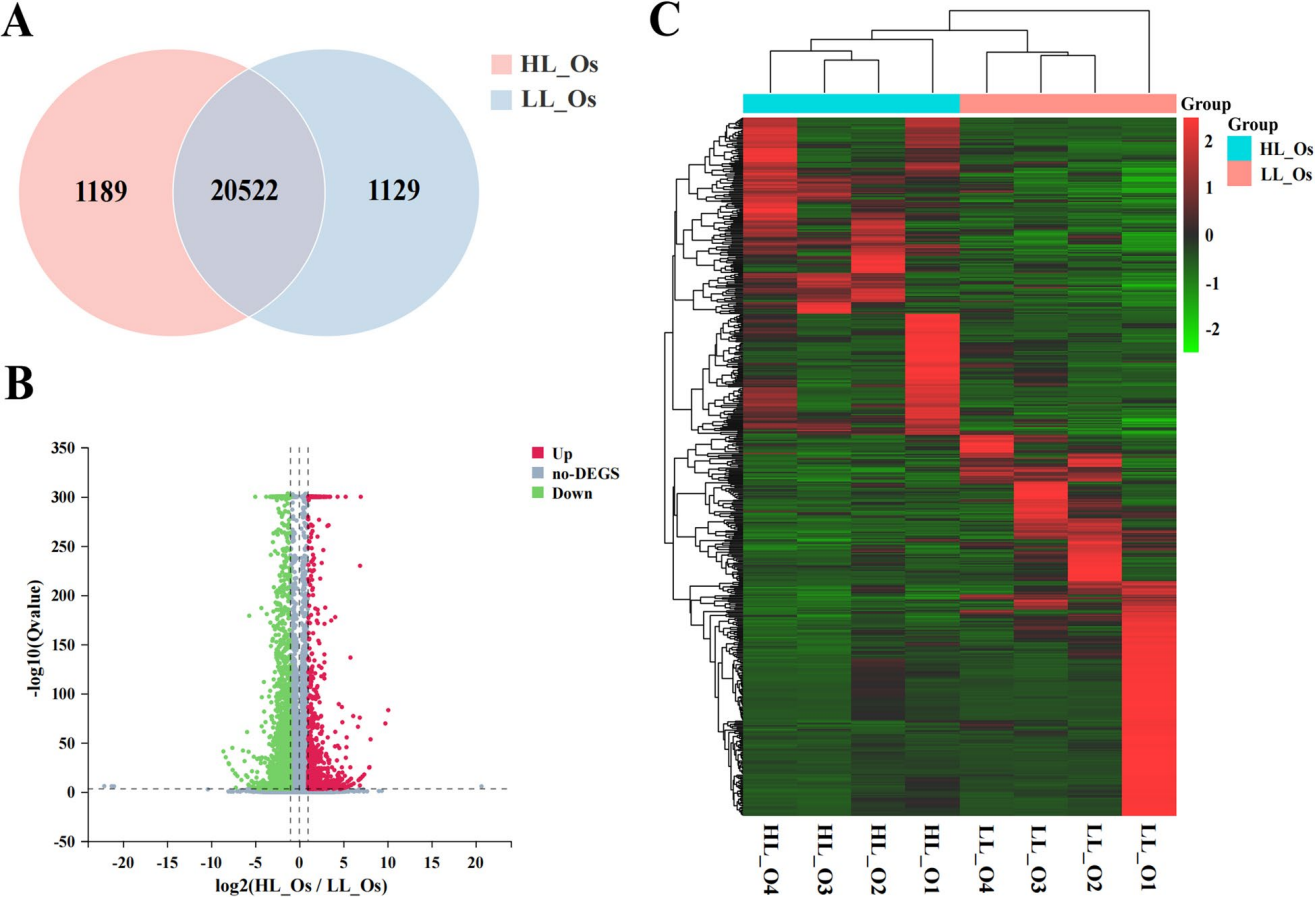
Screening results of DEGs in ovarian tissue: **A** Venn diagram of DEGs in ovarian tissue; **B** Volcano map of DEGs in ovarian tissue; **C** Heatmap of DEGs in ovarian tissue

**Fig. 2:**
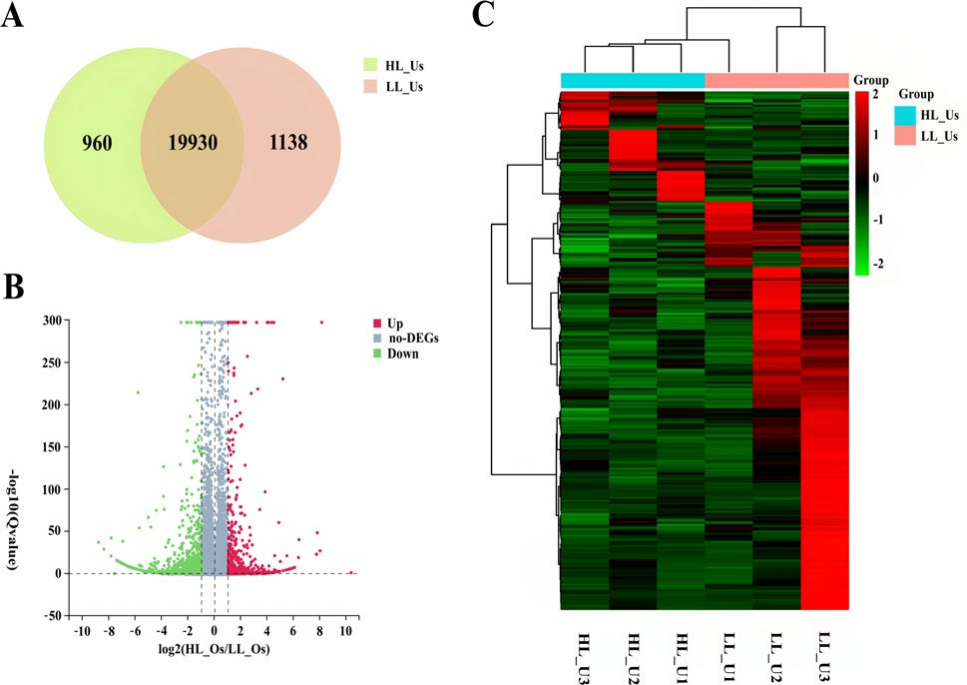
Genomic analysis results of the DEGs in the HL_Os and LL_Os groups: **A** Top 10 significantly enriched GO biological process terms; **B** KEGG pathway bubble diagram showing the top 30 significantly enriched pathways; **C** Diagram of KEGG pathways related to reproductive performance; **D** DEG protein interaction network diagram

Using HL_Os as the experimental group and LL_Os as the control group, 22,840 expressed genes were identified (Fig. 1A). On the basis of the threshold (|log2FC|≥1 and Q ≤ 0.05), 2,853 differentially expressed genes (DEGs) were selected (Fig. 1B). As shown in Fig. 1C, there were distinct expression patterns between the two groups, with significant differences in the expression of genes across each group. Gene Ontology (GO) and Kyoto Encyclopedia of Genes and Genomes (KEGG) enrichment analyses revealed that 352 GO terms were significantly enriched in 2,137 DEGs, and the enriched terms belonged primarily in the biological process (BP) category. To further investigate the functions of the DEGs, we also analysed the top 10 GO BP terms. As shown in Fig. 2A, six GO terms, including flagellated sperm motility, negative regulation of myoblast differentiation, branching involved in ureteric bud morphogenesis, locomotory behavior, inner dynein arm assembly and positive regulation of gene expression were significantly enriched in 13 genes related to prolificacy, including *ENO4*, *CXCL14*, *HOXA11*, *BCL2*, *DNAH*1, and *DRD1*. Additionally, multiple GO terms related to reproductive performance, including cell adhesion, hormone biosynthetic process, uterus development, and positive regulation of the Wnt signalling pathway, were enriched in these genes.

The KEGG enrichment analysis revealed that a total of 85 pathways were enriched in 1,053 DEGs. We used a P-value of < 0.05 as the criterion for screening, 63 significantly enriched pathways were selected. As shown in Fig. 2B, the top 30 enriched KEGG pathways were pathways associated with neuroactive ligand‒receptor interactions, viral protein interactions with cytokines and cytokine receptors, and cytokine‒cytokine receptor interactions. It also revealing that pathways related to reproductive performance, including the ovarian steroidogenesis pathway, the cAMP signalling pathway, pathways related to cell adhesion molecules, pathways related to ECM‒receptor interactions and the calcium signalling pathway were significantly enriched in genes such as *DRD1*, *BMP15*, *FSHR*, *WNT10B*, *3BHSD*, and *CYP19A1* (Fig. 2C). PPI analysis was subsequently conducted to construct a protein‒protein interaction network map, which revealed that proteins such as GABRA1, WNT10B, BEST4, CLCNKA, and CLIC6 play key roles (Fig. 2D).

### Screening and analysis of candidate genes related to prolificacy in uterine tissue

**Fig. 3:**
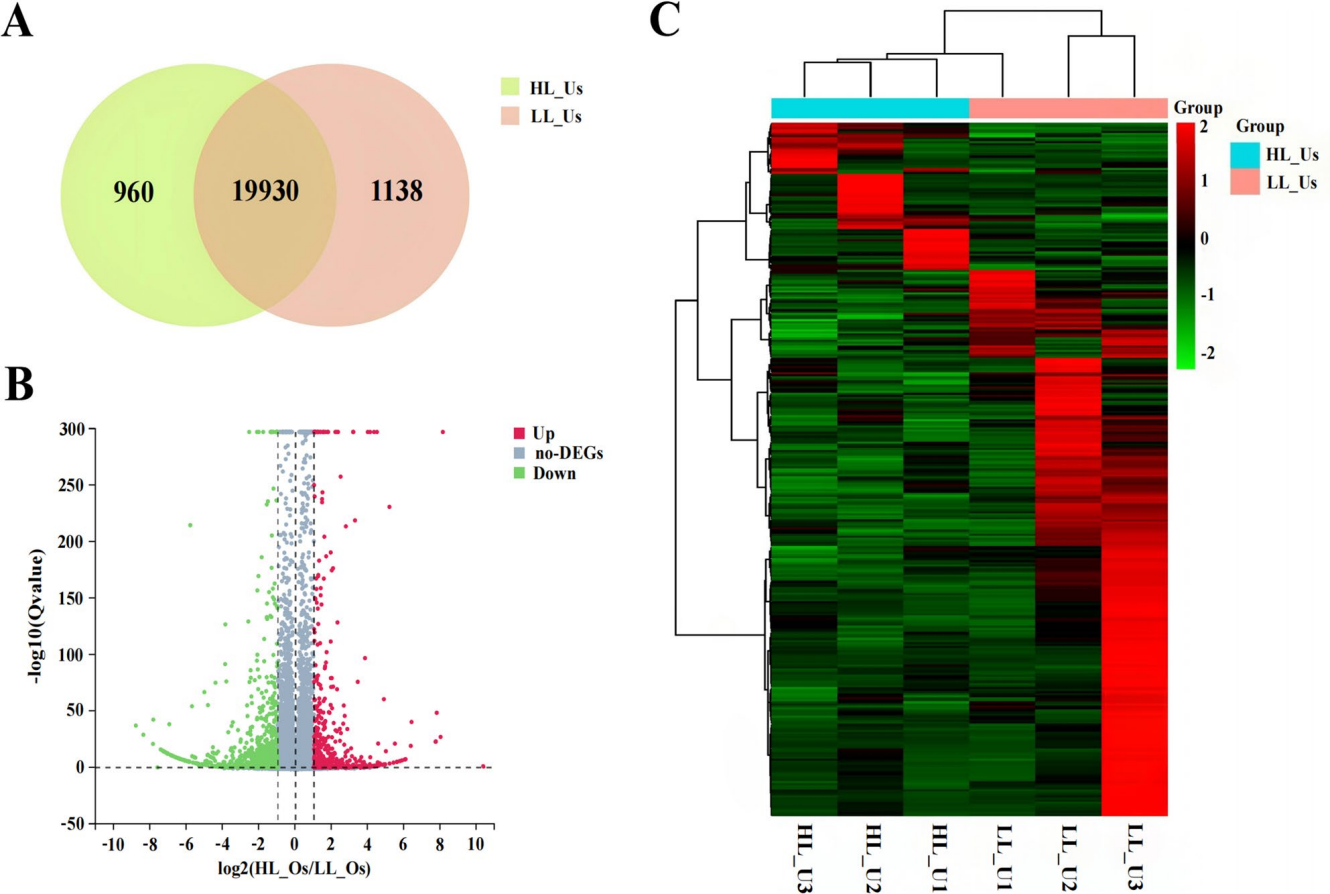
Screening results of DEGs in uterine tissue: **A** Venn diagram of DEGs; **B** Volcano plot of DEGs; **C** Heatmap of DEGs

**Fig. 4:**
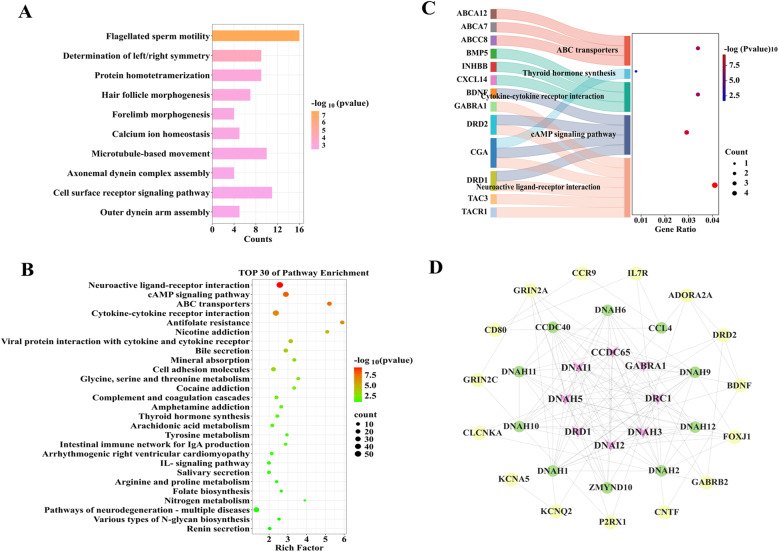
Genomic analysis results of the DEGs in the HL_Us and LL_Us group: **A** Top 10 significantly enriched GO BP terms; **B** Bubble plot showing the top 30 significantly enriched KEGG pathways; **C** KEGG pathways related to reproductive performance; **D** DEG protein interaction network

HL_Us were used as the experimental group, and LL_Us were used as the control group. A total of 22,028 expressed genes were identified in six uterine tissues (Fig. 3A). A total of 1,306 DEGs were selected on the basis of the criteria (|log2FC|≥1 and Q ≤ 0.05) (Fig. 3B). Cluster analysis was also conducted, and as shown in Fig. 3C, there were distinct expression patterns between the two groups, with significant differences in the expression of genes across groups. GO functional enrichment analysis revealed that 163 GO terms, primarily BP terms, were significantly enriched in 988 DEGs. Subsequently, the top 10 enriched BP terms, as shown in Fig. 4A, were subjected to GO Term analysis, which revealed that genes such as *3BHSD*, *ENO4*, *SYCP1*, *WNT10B*, *DNAH1*,* CFAP43*, and *FGF7*, which were significantly associated with GO terms related to forelimb morphogenesis, cell adhesion, and sperm axoneme assembly, were involved in the regulation of multiple reproductive traits.

The KEGG enrichment analysis revealed that a total of 28 pathways were significantly enriched in 479 DEGs. Using a significance threshold of *P* < 0.05, we identified 19 enriched pathways. As shown in Fig. 4B, the top 30 enriched KEGG pathways were pathways related to the neuroactive ligand‒receptor interaction, pathways related to ABC transporters, and folate resistance pathways. And we revealed that genes such as *TACR1*, *GABRA1*, *DRD1*, *CXCL14*, and *INHBB* were significantly associated with neuroactive ligand‒receptor interactions, the cAMP signalling pathway, and cytokine‒cytokine receptor interaction pathways related to reproductive performance (Fig. 4C). PPI analysis was subsequently conducted, and a PPI network was constructed (Fig. 4D), indicating that proteins such as DRD1, GABRA1, DNAH5, and CCDC65 play key roles.

###  Integration of candidate genes related to prolificacy identified in different tissues

**Fig. 5:**
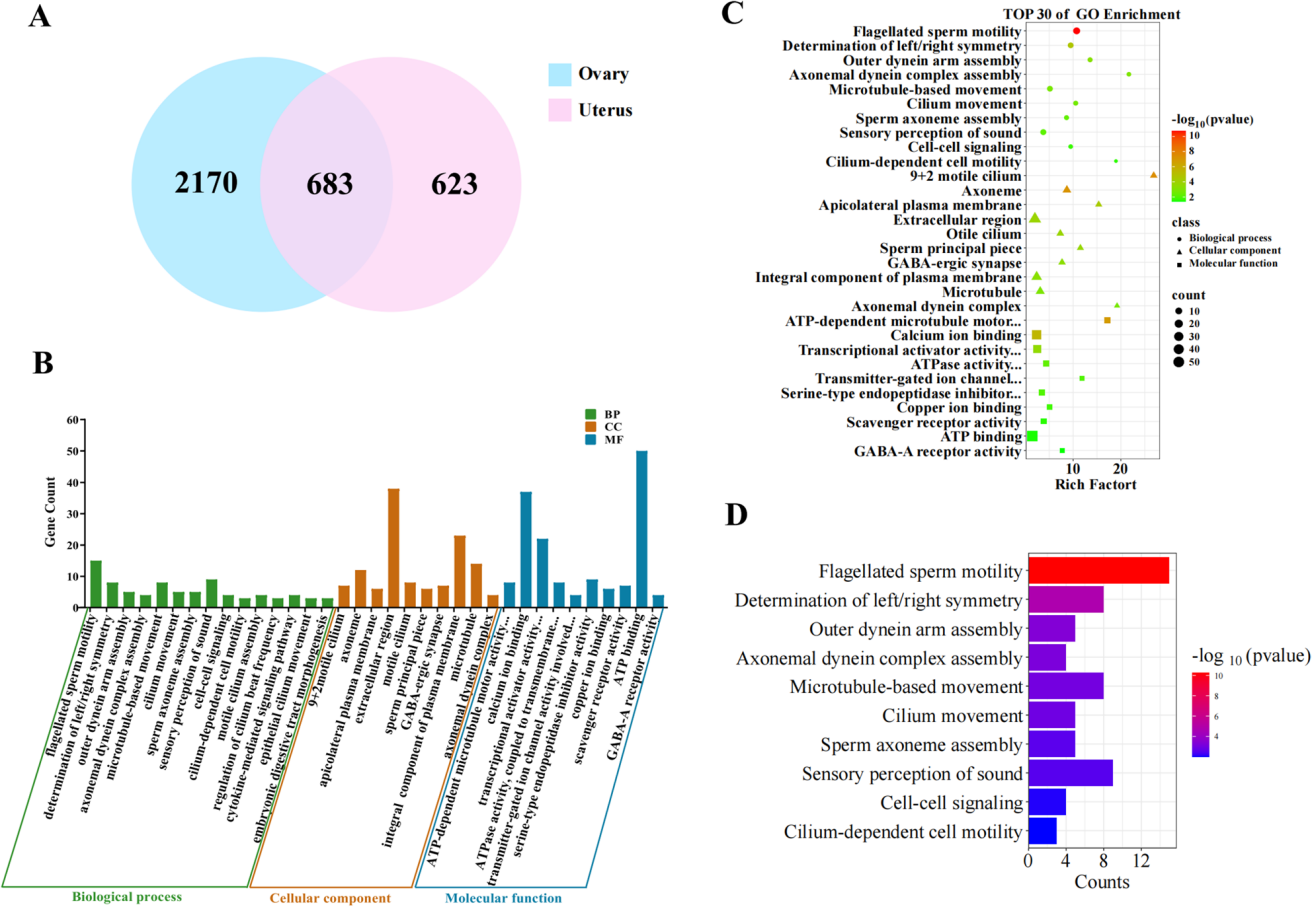
Screening of DEGs between different tissues and differential gene analysis: **A** Venn diagram of the DEGs; **B** GO function classification statistics; **C** Top 30 significantly enriched GO terms; **D** Top 10 significantly enriched GO BP terms

The samples were reclassified into two groups, the ovary group and the uterus group, and the sequencing data from both the ovaries and the uterus were integrated to analyse the DEGs in both groups. As shown in Fig. 5A, there were 683 genes expressed between the two groups, with 2170 specific DEGs in the ovary group and 623 specific DEGs in the uterus group. GO and KEGG enrichment analyses were subsequently performed on the 683 DEGs, revealing that 102 GO terms, primarily in the BP category, were enriched in 337 DEGs (*P* < 0.05) (Fig. 5B and C). To explore additional functional attributes of the DEGs, we also analysed the top 10 GO BP terms (Fig. 5D), identifying three candidate genes related to prolificacy, namely, *ENO4*, *DNAH1*, and *CFAP43*, which were associated with embryonic digestive tract morphogenesis, flagellar sperm motility, sperm axoneme assembly, and thyroid hormone generation, respectively. Additionally, we found that cell adhesion molecule entry is closely associated with reproductive performance.

**Fig. 6:**
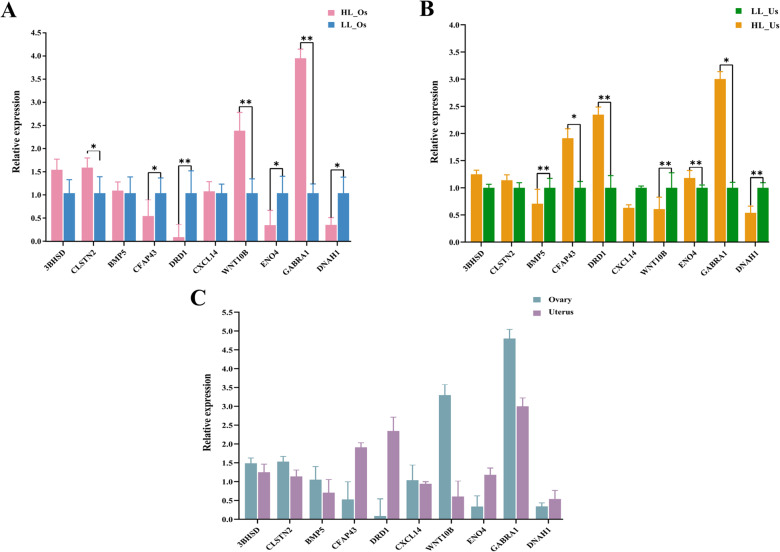
KEGG enrichment analysis results of the DEGs: **A** KEGG pathway function classification statistics; **B** Bubble diagram of the top 30 significantly enriched KEGG pathways; **C **String diagram of KEGG pathways related to reproductive traits; **D** DEG protein interaction network diagram

KEGG enrichment analysis revealed that 26 pathways were significantly enriched in 229 DEGs (Fig. 6A). The top 30 enriched KEGG pathways (all meeting the significance threshold of *P* < 0.05) included the cAMP signalling pathway, pathways related to the neuroactive ligand‒receptor interaction, and antifolate resistance pathways (Fig. 6B). From these, 17 significantly enriched pathways were selected. Genes such as *DRD1*, *DRD2*, *GRIA3*, *GABRA1*, *CXCL14*, *BMP5*, *ENO4*, and *3BHSD* were significantly associated with five pathways related to reproductive performance, including the cAMP signalling pathway, pathways related to the neuroactive ligand‒receptor interaction, and pathways related to cytokine‒cytokine receptor interactions (Fig. 6C).

PPI analysis was subsequently conducted on the common DEGs in ovarian and uterine tissues to construct a PPI network. As shown in Fig. 6D, proteins such as GABRA1, CCDC65, CFAP43, DNAH11, and WDR63 are at key positions within this network. These findings suggest that the genes *3BHSD*, *CLSTN2*, *BMP5*, *DRD1*,* CXCL14*, *CFAP43*, *WNT10B*, *ENO*4, *GABRA1*, and *DNAH1* may be associated with fecundity in Jining grey goats.

### Validation of DEGs

RT‒qPCR validation was conducted on 10 selected candidate genes. According to the amplification and melting curve results, all the gene amplification curves showed suitable overall parallelism and high amplification efficiency; the melting curves were single peaks, suggesting that there were no nonspecific amplification products (Supplementary Fig. 2). Comprehensive analysis revealed that the PCR system and reaction conditions used in the experiment were excellent, and the RT‒qPCR results were consistent with the RNA‒seq results, indicating that the RNA‒seq results of this study are accurate and reliable.

**Fig. 7:**
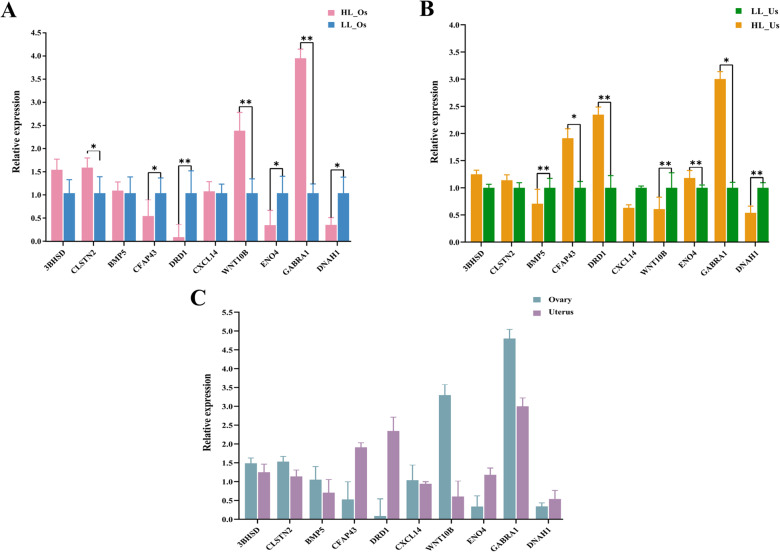
Comparison of the expression levels of 10 candidate genes: **A** Comparison of the expression levels of candidate genes in the HL_Os and LL_Os samples; **B** Comparison of the expression levels of candidate genes in the HL_Us and LL_Us samples; **C** Comparison of the expression levels of candidate genes in different tissues

The expression levels of 10 candidate genes were subsequently detected in the ovarian and uterine tissues of the HL and LL groups. As shown in Fig. 7A, the genes *3BHSD*, *CLSTN2*, *BMP5*,* CXCL14*, *WNT10B*, and *GABRA1* were highly expressed in the HL_Os group, with *CLSTN*2, *WNT10B*, and *GABRA1* showing significantly higher expression levels than those in the LL_Os group; the genes *CFAP43*, *DRD1*, *ENO4*, and *DNAH1* were highly expressed in the LL_Os group, with significantly higher expression levels than those in the HL_Os group. As shown in Fig. 7B, the genes *3BHSD*, *CLSTN2*, *CFAP43*, *DRD1*, *ENO4*, and *GABRA1* were highly expressed in the HL_Us group, with *CFAP43*, *DRD1*, *ENO4*, and *GABRA1* showing significantly higher expression levels than those in the LL_Us group; the genes *BMP5*, *CXCL14*, *WNT10B*, and *DNAH1* were highly expressed in the LL_Us group, with *BMP5*, *WNT10B*, and *DNAH1* showing extremely significantly higher expression levels than those in the HL_Us group. Therefore, these 10 DEGs may collectively participate in the regulation of reproductive traits in ovarian and uterine tissues, making them important candidate genes for prolificacy in Jining grey goats.

A comparison of the relative expression levels of 10 candidate genes in different tissues revealed that these genes exhibited significant tissue-specific expression patterns. As shown in Fig. 7C, the expression levels of *3BHSD*, *CLSTN2*, *BMP5*, *CXCL14*, *WNT10B*, and *GABRA1* were high in the HL_Os, especially the expression levels of *WNT10B* and *GABRA1* in the HL_Os, which were much higher than those in the HL_Us. Moreover, the expression levels of *CFAP43*, *DRD1*, *ENO4*, and *DNAH1* were all greater in the HL_Us than those in the HL_Os. In summary, the genes *3BHSD*, *CLSTN2*, and *GABRA1* are highly expressed in both the HL_Os and HL_Us of Jining grey goats. These results provide foundational data for further investigation into the roles of these genes in regulating high reproductive processes in this goat breed.

## Discussion

The ovaries are vital organs in mammals and are responsible for key functions, such as follicle formation and hormone secretion. Moreover, the uterus, another important reproductive organ, is regulated by reproductive hormones, cytokines, and growth factors and serves as a crucial site for foetal growth and development [15–18]. Reproductive traits are complex traits influenced by both multiple genes and environmental factors. The ovulation rate of the ewe primarily determines lamb production, making it challenging to identify candidate genes for fecundity using traditional molecular biology techniques [19]. We compared the differential expression of these candidate genes across different tissues to characterize their tissue-specific expression patterns and provide foundational insights into their potential molecular regulatory mechanisms. Specifically, we identified five key pathways through GO and KEGG analyses that are potentially critical for reproductive regulation in Jining grey goats. These include the cAMP signalling pathway (regulating follicle maturation and ovulation [20, 21]), pathways related to the neuroactive ligand‒receptor interaction (involved in hypothalamic-pituitary-gonadal axis regulation [22, 23]), pathways related to ABC transporters (associated with steroid hormone transport [24]), pathways related to cytokine‒cytokine receptor interactions (crucial for maintaining the uterine microenvironment [25, 26]), and metabolic pathways (which provide energy support for reproductive processes [27]).

Steroid hormones are secreted by the ovaries of mammals and play crucial roles in regulating the endocrine system and maintaining normal reproduction [19]. In this study, the ovarian steroidogenesis pathway, which may be related to the lambing rate of Jining grey goats, was enriched in genes such as *3BHSD*, *BMP5*, and *FSHR*. Moreover, the bone morphogenetic protein (BMP) family promotes follicle growth and development in the ovary and is one of the main gene families affecting the reproductive performance of sheep and goats, playing a significant regulatory role in ovulation and lambing rates [28, 29]. Among these genes, the *BMP15*,* GDF9*, and *BMP5* genes, which are important members of the bone morphogenetic protein family, have been proven to be associated with prolificacy [30, 31]. High expression of the BMP15 gene can promote oocyte development and granulosa cell proliferation and differentiation while also playing a crucial role in regulating steroid hormones and ovulation [12]. Zhang et al. reported that the relative expression levels of the *GDF9* and *BMP15* genes were highest in the ovaries and lowest in the uteri of highly prolific Qianbei maroon goats, indicating that these two genes may play a significant regulatory role in the high prolificacy of Qianbei maroon goats [32]. The *BMP5* gene was initially discovered during bone development, and recent research on the role of *BMP5* in mammalian reproductive performance is limited and has focused mainly on its role in promoting apoptosis and treating cancer [33]. Pierre et al. reported that the *BMP5* gene can promote the proliferation of granulosa cells in rat ovaries and inhibit the synthesis and secretion of progesterone [34]. Zhong et al. screened 47 candidate genes that may be associated with the reproductive performance of sheep, including *BMP5*, *BMP15*, and *BMPR1B*. However, owing to the relatively low expression of *BMP5* in the ovaries and pituitary gland, further research [35] is still needed. Although there have been no reports on the genetic effects of the *BMP5* gene on the birth rates of Jining grey goats, the *BMP5* and *BMP15* genes were identified in the HL_Os group of Jining grey goats in this study. Therefore, more research is needed to elucidate the relationships between the expression patterns and functions of these genes.

The Wnt signalling pathway, which is mediated by the Wnt protein family, regulates processes such as cell proliferation, differentiation, migration, and apoptosis. These pathways can be divided into the classical Wnt signalling pathway, the Wnt/PCP pathway, and the Wnt/Ca2 + pathway. The classical Wnt signalling pathway plays a crucial role in embryonic development by regulating the proliferation and differentiation of epithelial mesenchymal cells [36, 37]. As an important member of the Wnt family, the *WNT2* gene promotes the growth and development of follicular granulosa cells through the classical Wnt signalling pathway, positively regulating ovarian development [38]. The WNT4 gene, as a regulatory factor, plays a significant role in mammalian reproductive development and ovarian function [39]. In this study, the *WNT2*, *WNT4*, and *WNT10B* genes, which encode proteins involved in the Wnt signalling pathway, were found to be associated with reproductive performance.

Zhang et al. collected uteri from pregnant Saanen dairy goats on days 5 and 15 of pregnancy to investigate the mechanisms of early pregnancy. They performed RNA-seq on these uteri and reported that the molecular mechanisms involved energy metabolism and amino acid metabolism, among other biological processes and pathways. They also hypothesized that genes such as *CXCL14*, *IGFBP3*, and *LGALS15* may promote uterine development and have beneficial effects throughout pregnancy [40]. Cell adhesion molecules are a class of transmembrane glycoproteins synthesized by cells that mediate intercellular adhesion within the ovary or between cells and the extracellular matrix and participate in related signal transduction, regulating follicle growth and development and thereby affecting reproductive performance in livestock [41–43]. In this study, the *HES1* gene, a cell adhesion molecule, was associated with prolificacy in the Jining grey goat. The cAMP signalling pathway controls various cellular processes and participates in cell proliferation and differentiation. When Yang et al. analysed candidate genes influencing lamb number in small-tailed Han sheep, they discovered through transcriptomics that the cAMP signalling pathway plays a role in reproductive performance, which is consistent with the results of this study [44].

In this study, the cAMP signalling pathway, metabolic pathways, cell adhesion molecules, and genes such as *3BHSD*, *CLSTN2*, *DRD1*, and *GRIA3* were revealed to be associated with reproductive performance. Moreover, the genes *3BHSD*, *CLSTN2*, and *GRIA3* were highly expressed in both the HL_Os and HL_Us groups, suggesting that their regulatory mechanisms may promote an increase in the lambing rate. Previous studies have indicated a close relationship between steroid and lipid metabolism and animal fertility [45], but no research has explicitly linked these factors to the reproductive performance of goats. Therefore, the metabolic pathways enriched in this study, which are related to reproductive performance, may provide new insights into the important functions of genetic diversity in goats, warranting further in-depth research on their mechanisms of action. Notably, the *3BHSD* gene has previously been shown to be significantly overexpressed in Jintang black goats with high prolificacy, and its differential expression between high- and low-little-size breeds aligns with the results of this study [46]. Recent studies have also demonstrated that genes related to sperm axoneme assembly play crucial roles in mammalian reproduction, such as the *CFAP43* and *DNAH1* genes, which are widely expressed in various tissues and organs, including the ovaries and uterus, influencing reproductive traits in mammals [47, 48]. Research has confirmed that the *CFAP43* and *DNAH1* genes are closely related to reproductive performance in various species, including humans, mice, cattle, and sheep. However, there are no studies on the relationships between the *CFAP43* and *DNAH1* genes and the prolificacy of Jining grey goats. In this study, the *CFAP43* gene was highly expressed in the LL_Os group and HL_Us group and had tissue-specific expression. Therefore, investigating the role of the *CFAP43* and *DNAH1* genes is highly important for the breeding of animals with high prolificacy.

## Conclusion

In this study, sequencing data from the ovaries and uterus were integrated and 5 key regulatory pathways (the cAMP signalling pathway, pathways associated with the neuroactive ligand‒receptor interaction, pathways associated with ABC transporters, pathways associated with cytokine‒cytokine receptor interactions and metabolic pathways) and 10 candidate genes (*3BHSD*, *CLSTN2*, *BMP5*, *DRD1*, *CXCL14*, *CFAP43*, *WNT10B*, *ENO4*, *GABRA1* and the *DNAH1* gene) were revealed to be associated with prolificacy in Jining grey goats. This study revealed that these 10 genes exhibit significant tissue specificity in ovarian and uterine tissues, with *3BHSD*, *CLSTN2*, and *GABRA1* being closely related to prolificacy in Jining grey goats. In summary, we identified the key regulatory pathways and candidate genes associated with prolificacy in Jining grey goats, providing insights into the molecular mechanisms by which candidate genes regulate prolificacy at the transcriptome level. These findings provide a theoretical foundation and reference for the selective breeding of Jining grey goats.

## Methods and materials

### Selection of animals and organization preparation

The experimental animals were obtained from the National Jining Grey Goat Breeding Farm in Jiaxiang, Jining, Shandong Province, China. Eight healthy adult ewes of consistent age and weight were selected and maintained under standard management conditions. The experimental animals were divided into two groups: the high litter size group (HL, litter size ≥ 3 in three consecutive litters, *n* = 4) and the low litter size group (LL, litter size = 1 in three consecutive litters, *n* = 4). We used vaginal implantation of CIDR suppositories to induce synchronized oestrous in ewes. We implanted CIDR inserts for 14 days, removed them, and then administered 300 IU pregnant mare serum gonadotropin (PMSG) intramuscularly. The oestrus status of the ewes was determined via vaginal examination. The ovaries (*n* = 8) and uterus (*n* = 6) were collected within 24 h after oestrous onset and stored in a −80 °C ultralow temperature freezer until total RNA extraction.

### Total RNA extraction

TRIzol reagent (Invitrogen, Carlsbad, CA, USA) was used to extract total RNA from the ovarian and uterine tissues. The integrity of the RNA was determined by agarose gel electrophoresis, and the concentration and purity of the RNA were measured using a NanoDrop™ One spectrometer (Thermo Scientific, Waltham, MA, USA) to ensure that the samples met the standards. Finally, the total RNA was stored at −80 °C.

### Transcriptome library construction and sequencing

CDNA libraries were constructed at BGI Genomics Co., Ltd. in China, and PE 100 bp dual-end sequencing was performed on the DNBSEQ platform. Using SOAPnuke (v1.5.6) software [49], reads with rRNA contamination, adapter contamination, low quality, and high N content were filtered to obtain clean reads. HISAT2 (v2.1.0) software [50] was then used to align the clean reads to the reference genome sequence (GCF_001704415.1_ARS1) of *Capra hircus*.

### Differential expression analysis

RSEM (v1.3.1) [51] software was used for gene expression quantification, and pheatmap (v1.0.8) was used to create heatmaps of the gene expression profiles of different samples. Three biological replicates were included for each sample, and the threshold criteria for selecting differentially expressed genes (DEGs) was a |log2(fold change)| ≥ 1 and a Q value (P-adjust) ≤ 0.05. DEGs were screened using DESeq2 software. Additionally, volcano plots and scatter plots were created to illustrate the upregulation and downregulation of the DEGs.

### Bioinformatics analysis

DAVID 6.8 software [52] was used to perform Gene Ontology (GO) and Kyoto Encyclopedia of Genes and Genomes (KEGG) pathway analyses, and the threshold was set at *P* < 0.05 to screen out significantly enriched GO terms and KEGG pathways. The Pearson correlation coefficients between proteins were analysed using the STRING online database, and the data were imported into Cytoscape v3.9.1 for visualization. The MCC algorithm was subsequently applied to analyse the protein interaction relationships among the DEGs, construct a protein‒protein interaction (PPI) network and screen candidate genes.

### Real-time fluorescence quantitative verification

Total RNA was extracted from early-stage ovarian and uterine samples. cDNA synthesis was performed using a PrimeScript RT Reagent Kit (Takara Bio Inc. Shiga, Japan). Primers were designed and synthesized by Shanghai Sangon Biotech Co., Ltd., on the basis of the mRNA sequences of various genes provided on the NCBI website. GAPDH was used as the internal reference gene, and quantitative PCR experiments were conducted using TB Green^®^ Premix Ex Taq™ I (Takara Bio Inc. Shiga, Japan), with RNA-seq data for comparative validation. Finally, the relative expression levels of the DEGs were calculated using the 2-ΔΔCt method. All experiments were repeated three times, and the data are expressed as the mean ± standard error of the mean (SEM). The primer sequences are shown in Table 1.

**Table 1:** Primer sequences

Gene	Primer (5’−3’)	Tm (°C)	Product length (bp)
*3BHSD*	F: CTCAGACGACACACCACACCAAAG R: CAGCAGGAAGGCAAGCCAGTAC	60	127
*CLSTN2*	F: GCCCACCAGCACTTCATCCAAG R: CTCCTCTTCCTCGGTCTCCTCTTC	60	144
*BMP5*	F: GACTCCTCCAGAATGTCCAGTGTTG R: GTGCTATAATCCAGTCCTGCCATCC	60	121
*DRD1*	F: GCCTGTTTCCTGTCTCTGCTCATC R: CACCAAGAGATCCGATACAGCCAAG	60	141
*CXCL14*	F: GAAAGGGACCCAAGATTCGCTACAG R: GATAACCATCTTCTCCTCGCAGTGC	60	86
*CFAP43*	F: TGAACGAGGGAGGCTGAGAGATG R: TCAAGACCGCAGAGGACAGAGTG	60	80
*WNT10B*	F: GTCTCCTGTTCCTGGCGTTGTG R: GCAGACTGTGTTGGCGGTCAG	60	104
*ENO4*	F: GCAGCAGGCGATGGAGTATTACC R: TGGAGGTAGAAGGTGGAGTTGAGC	60	81
*GABRA1*	F: TCTGAGCACATTGACTGGAAGAAGC R: AATCCTGGTGAAGACAGTGGTGTTG	60	85
*DNAH1*	F: AGCACCTCAGCAACCTCTCCAG R: GGGCACCTTCTCCTCCTCCTTC	60	143
*GAPDH*	F: CGGCACAGTCAAGGCAGAGAAC R: CACGTACTCAGCACCAGCATCAC	60	115

## Supplementary Information

Supplementary Material 1.

Supplementary Material 2.

## Data Availability

All RNA-seq data generated in this study were submitted to the NCBI SRA database under BioProject No. PRJNA1068677 and No. PRJNA1347776.

## Abbreviations

NCBI: National Center for Biotechnology Information
HL: High litter size
LL: Low litter size
HL_OS: High litter size ovaries
LL_OS: Low litter size ovaries
HL_Us: High litter size uterus
LL_Us: Low litter size uterus
DEGs: Differentially expressed genes
RNA-seq: Ribonucleic acid sequencing
PCA: Principal component analysis
PCR: Polymerase chain reaction
RT‒qPCR: Reverse transcription‒quantitative PCR
GO: Gene Ontology
KEGG: Kyoto Encyclopedia of Genes and Genomes
PPI: Protein–protein interaction
cAMP: Cyclic adenosine monophosphate
Wnt: Wingless/Integrated
ABC: ATP-binding cassette
ECM: Extracellular matrix
ABC: ATP-binding cassette
GWAS: Aenome-Wide Association Studies
BP: Biological processes
BMP: Bone Morphogenetic Protein

## References

[1] Wang JJ, Zhang T, Chen QM, Zhang RQ, Li L, Cheng SF, et al. Genomic signatures of selection associated with litter size trait in Jining Gray goat. Front Genet. 2020;11:286. 32273886 10.3389/fgene.2020.00286 PMC7113370

[2] Ajafar MH, Kadhim AH, Al-Thuwaini TM. The reproductive traits of sheep and their influencing factors. RAS. 2022;10:82–9.

[3] Nosrati M, Asadollahpour NH, Amiri GZ, Esmailizadeh A. Whole genome sequence analysis to detect signatures of positive selection for high fecundity in sheep. Reprod Domest Anim. 2019;54(2):358–64. 30359467 10.1111/rda.13368

[4] Mao W, Wang P, Zhou L, Li D, Li X, Lou X, Huang X, Wang F, Zhang Y, Liu J, Wan Y. An upgraded nuclease prime editor platform enables high-efficiency singled or multiplexed knock-in/knockout of genes in mouse and sheep zygotes. Protein Cell. 2025;16(8):732–8. 10.1093/procel/pwaf006 PMC12342182 39832212

[5] Cai Y, Xu H, Deng K, Yang H, Zhao B, Zhang C, Li S, Wei Z, Wang Z, Wang F, Zhang Y. A novel nuclear receptor NR1D1 suppresses HSD17B12 transcription to regulate granulosa cell apoptosis and autophagy via the AMPK pathway in sheep. Int J Biol Macromol. 2025;306(01):141271. 39986531 10.1016/j.ijbiomac.2025.141271

[6] McKey J, Anbarci DN, Bunce C, Ontiveros AE, Behringer RR, Capel B. Integration of mouse ovary morphogenesis with developmental dynamics of the oviduct, ovarian ligaments, and rete ovarii. Elife. 2022;11:e81088. 36165446 10.7554/eLife.81088 PMC9621696

[7] Pan B, Chai J, Fei K, Zheng T, Jiang Y. Dynamic changes in the transcriptome and metabolome of pig ovaries across developmental stages and gestation. BMC Genomics. 2024;25(1):1193. 39695358 10.1186/s12864-024-11122-3 PMC11654078

[8] Zhang J, Xia W, Zhou J, Qin S, Lin L, Zhao T, et al. Participation of preovulatory follicles in the activation of primordial follicles in mouse ovaries. Int J Biol Sci. 2024;20(10):3863–80. 39113716 10.7150/ijbs.95020 PMC11302884

[9] Shokrollahi B, Morammazi S. Polymorphism of GDF9 and BMPR1B genes and their association with litter size in Markhoz goats. Reprod Domest Anim. 2018;53(4):971–8. 29696699 10.1111/rda.13196

[10] Wang JQ, Cao WG. Progress in exploring genes for high fertility in ewes. Yi Chuan. 2011;33(9):953–61. 21951796 10.3724/sp.j.1005.2011.00953

[11] Ma X, Liu A, Tian S. A meta-analysis of mRNA expression profiling studies in sheep with different FecB genotypes. Anim Genet. 2023;54(3):225–38. 36811249 10.1111/age.13304

[12] Yang Z, Yang X, Liu G, Deng M, Sun B, Guo Y, et al. Polymorphisms in BMPR-IB gene and their association with litter size trait in Chinese Hu sheep. Anim Biotechnol. 2022;33(2):250–9. 32657205 10.1080/10495398.2020.1789158

[13] Su P, Gu Y, Wang S, Cao X, Lv X, Getachew T, et al. FecB was associated with litter size and follows mendel’s laws of inheritance when it transited to next generation in Suhu meat sheep breeding population. Genes. 2024;15(3):260. 38540319 10.3390/genes15030260 PMC10970568

[14] Sun X, Niu Q, Jiang J, Wang G, Zhou P, Li J, et al. Identifying candidate genes for litter size and three morphological traits in Youzhou dark goats based on genome-wide SNP markers. Genes. 2023;14(6):1183. 37372363 10.3390/genes14061183 PMC10298679

[15] Zhu Z, He M, Zhang T, Zhao T, Qin S, Gao M, et al. LSD1 promotes the FSH responsive follicle formation by regulating autophagy and repressing Wt1 in the granulosa cells. Sci Bull (Beijing). 2024;9(8):1122–36. 10.1016/j.scib.2024.01.015 38302330

[16] Hong L, Zang X, Hu Q, He Y, Xu Z, Xie Y, Gu T, Yang H, Yang J, Shi J, Zheng E, Huang S, Xu Z, Liu D, Cai G, Li Z, Wu Z. Uterine luminal-derived extracellular vesicles: potential nanomaterials to improve embryo implantation. J Nanobiotechnol. 2023;1(1):79. 10.1186/s12951-023-01834-1 PMC9990359 36882792

[17] Strączyńska P, Papis K, Morawiec E, Czerwiński M, Gajewski Z, Olejek A, et al. Signaling mechanisms and their regulation during in vivo or in vitro maturation of mammalian oocytes. Reprod Biol Endocrinol. 2022;20(1):37. 35209923 10.1186/s12958-022-00906-5 PMC8867761

[18] Han B, Tian D, Li X, Liu S, Tian F, Liu D, et al. Multiomics analyses provide new insight into genetic variation of reproductive adaptability in Tibetan sheep. Mol Biol Evol. 2024;41(3):msae058. 38552245 10.1093/molbev/msae058 PMC10980521

[19] Liu JC, Xing CH, Xu Y, Pan ZN, Zhang HL, Zhang Y, et al. DEHP exposure to lactating mice affects ovarian hormone production and antral follicle development of offspring. J Hazard Mater. 2021;416:125862. 34492810 10.1016/j.jhazmat.2021.125862

[20] De HT, Si JL, Fei T, et al. Comparative transcriptome of reproductive axis in Chinese Indigenous sheep with different FecB genotypes and prolificacies. Anim Reprod Sci. 2020;223:106624. 33126044 10.1016/j.anireprosci.2020.106624

[21] Xiao X, Ji Z, Wang T, et al. Investigation of high fecundity genes by nanopore sequencing in sheep (*Ovis aries*) pituitary. BMC Genomics. 2025;26(1):564. 40474086 10.1186/s12864-025-11732-5 PMC12143060

[22] Wei S, Kang X, Yang C, et al. Analysis of reproduction-related transcriptomes on pineal-hypothalamic-pituitary-ovarian tissues during estrus and anestrus in Tan sheep. Front Vet Sci. 2022;24(9):1068882. 10.3389/fvets.2022.1068882 PMC9729709 36504859

[23] Li Q, Chao T, Wang Y, et al. The transcriptome characterization of the hypothalamus and the identification of key genes during sexual maturation in goats. Int J Mol Sci. 2024;25(18):10055. 39337542 10.3390/ijms251810055 PMC11432450

[24] Sales AD, Duarte ABG, Rocha RMP, et al. Transcriptional downregulation of ABC transporters is related to follicular degeneration after vitrification and in vitro culture of ovine ovarian tissue. Theriogenology. 2022;177:127–32. 34700069 10.1016/j.theriogenology.2021.10.013

[25] Ji T, Chen X, Zhang Y, et al. Effects of N-acetylcysteine on the proliferation, hormone secretion level, and gene expression profiles of goat ovarian granulosa cells. Genes. 2022;13(12):2306. 36553574 10.3390/genes13122306 PMC9778279

[26] Deng K, Li X, Liu Z, et al. IGF2BP2 regulates the proliferation and migration of endometrial stromal cells through the PI3K/AKT/mTOR signaling pathway in Hu sheep. J Anim Sci. 2024;102:129. 10.1093/jas/skae129 PMC11151927 38727196

[27] Lebachelier de la Lebachelier ME, Téteau O, Mahé C, et al. Metabolic status is a key factor influencing proteomic changes in Ewe granulosa cells induced by chronic BPS exposure. BMC Genomics. 2024;25(1):1095. 39550580 10.1186/s12864-024-11034-2 PMC11568600

[28] Wang J, Hao Z, Hu L, Qiao L, Luo Y, Hu J, et al. MicroRNA-199a-3p regulates proliferation and milk fat synthesis of ovine mammary epithelial cells by targeting VLDLR. Front Vet Sci. 2022;9:948873. 35990270 10.3389/fvets.2022.948873 PMC9391033

[29] Miao X, Luo Q. Genome-wide transcriptome analysis between small-tail Han sheep and the Surabaya fur sheep using high-throughput RNA sequencing. Reproduction. 2013;145(6):587–96. 23579189 10.1530/REP-12-0507

[30] Torrecilha RBP, Milanesi M, Wade CM, Gallana M, Falbo AK, Reichler IM, Hug P, Jagannathan V, Trigo BB, Paulan SC, Bruno DB, Garcia SD, Scaramele NF, Lopes FL, Dolf G, Leeb T, Sölkner J, Garcia JF, Pieńkowska-Schelling A, Schelling C, Utsunomiya YT. Association of missense variants in GDF9 with litter size in entlebucher mountain dogs. Anim Genet. 2020;51(1):78–86. 31802524 10.1111/age.12882

[31] Das A, Shaha M, Gupta MD, Dutta A, Miazi OF. Polymorphism of fecundity genes(BMP15 and GDF9)and their association with litter size in Bangladeshi prolific black Bengal goat. Trop Anim Health Prod. 2021;53(2):1–8. 10.1007/s11250-021-02679-2 33772358

[32] Zhang Y, Chen X, Ruan Y, Guo W, Chen J, Tang W, Ji Q, Fu K. Effect of CTSS non-synonymous mutations on litter size in Qianbei Ma goats. Front Vet Sci. 2023;27(10):1276673. 10.3389/fvets.2023.1276673 PMC10711060 38089704

[33] Weaver S, Shank SD, Spielman SJ, Li M, Muse SV, Kosakovsky Pond SL. Datamonkey 2.0: a modern web application for characterizing selective and other evolutionary processes. Mol Biol Evol. 2018;35(3):773–7. 29301006 10.1093/molbev/msx335 PMC5850112

[34] Pierre A, Pisselet C, Dupont J, Bontoux M, Monget P. Bone morphogenetic protein 5 expression in the rat ovary: biological effects on granulosa cell proliferation and steroidogenesis. Biol Reprod. 2009;73(6):1102–8. 10.1095/biolreprod.105.043091 16079308

[35] Chong YQ, Liu GQ, Jiang XP. Effect of BMPR1B gene on litter size of sheep inChina: a meta-analysis. Anim Reprod Sci. 2019;210:106175. 31635771 10.1016/j.anireprosci.2019.106175

[36] Li Q, Shi J, Liu W. The role of Wnt/β-catenin-lin28a/let-7 axis in embryo implantation competency and epithelial-mesenchymal transition(EMT). Cell Commun Signal. 2020;18(1):1–13. 32650795 10.1186/s12964-020-00562-5 PMC7353806

[37] Mosimann C, Hausmann G, Basler K. Beta-catenin hits chromatin: regulation of Wnt target gene activation. Nat Rev Mol Cell Biol. 2009;10:276–86. 19305417 10.1038/nrm2654

[38] Miyoshi T, Otsuka F, Inagaki K, Otani H, Takeda M, Suzuki J, et al. Differential regulation of steroidogenesis by bone morphogenetic proteins in granulosa cells: involvement of extracellularly regulated kinase signaling and oocyte actions in follicle-stimulating hormone-induced estrogen production. Endocrinology. 2007;148(1):337–45. 17008391 10.1210/en.2006-0966

[39] Babaki S, Zavareh S, Farrokh P, Nasiri M. Evaluating the expression of Wnt pathway related genes in mouse vitrified preantral follicles: an experimental study. J Reprod Infertil. 2021;22(3):151–8. 34900635 10.18502/jri.v22i3.6715 PMC8607873

[40] Zhang L, Liu X, Liu J, Ma L, Zhou Z, Song Y, et al. The developmental transcriptome landscape of receptive endometrium during embryo implantation in dairy goats. Gene. 2017;633:82–95. 28866083 10.1016/j.gene.2017.08.026

[41] Shen J, Zhao W, Cheng J, Cheng J, Zhao L, Dai C, et al. Lipopolysaccharide accelerates tryptophan degradation in the ovary and the derivative kynurenine disturbs hormone biosynthesis and reproductive performance. J Hazard Mater. 2023;458:131988. 37418963 10.1016/j.jhazmat.2023.131988

[42] Zhang H, Li C, Wen D, Li R, Lu S, Xu R, et al. Melatonin improves the quality of maternally aged oocytes by maintaining intercellular communication and antioxidant metabolite supply. Redox Biol. 2022;49:102215. 34929573 10.1016/j.redox.2021.102215 PMC8688718

[43] Wu HY, Ji ZH, Xie WY, Guo HX, Zheng Y, Gao W, et al. KLF4 promotes milk fat synthesis by regulating the PI3K-AKT-mTOR pathway and targeting FASN activation in bovine mammary epithelial cells. iScience. 2024;27(6):109850. 38779481 10.1016/j.isci.2024.109850 PMC11108978

[44] Yang J, Tang J, He X, Di R, Zhang X, Zhang J, et al. Key mRNAs and LncRNAs of pituitary that affect the reproduction of FecB + + small tail Han sheep. BMC Genomics. 2024;25(1):392. 38649819 10.1186/s12864-024-10191-8 PMC11034058

[45] Chou CH, Chen MJ. The effect of steroid hormones on ovarian follicle development. Vitam Horm. 2018;107:155–75. 29544629 10.1016/bs.vh.2018.01.013

[46] Zi XD, Lu JY, Zhou H, Ma L, Xia W, Xiong XR, Lan DL, Wu XH. Comparative analysis of ovarian transcriptomes between prolific and non-prolific goat breeds via high-throughput sequencing. Reprod Domest Anim. 2018;53(2):344–51. 29134700 10.1111/rda.13111

[47] Coutton C, Vargas AS, Amiri-Yekta A, Kherraf ZE, Ben Mustapha SF, Le Tanno P, et al. Mutations in CFAP43 and CFAP44 cause male infertility and flagellum defects in *trypanosoma* and human. Nat Commun. 2018;9(1):686. 29449551 10.1038/s41467-017-02792-7 PMC5814398

[48] Kang Z, Jiang E, Wang K, Pan C, Chen H, Yan H, et al. Goat membrane associated ring-CH-type finger 1 (MARCH1) m RNA expression and association with litter size. Theriogenology. 2019;128:8–16. 30711644 10.1016/j.theriogenology.2019.01.014

[49] Cock PJ, Fields CJ, Goto N, Heuer ML, Rice PM. The Sanger FASTQ file format for sequences with quality scores and the Solexa/Illumina FASTQ variants. Nucleic Acids Res. 2010;38(6):1767–71. 20015970 10.1093/nar/gkp1137 PMC2847217

[50] Kim D, Langmead B, Salzberg SL. HISAT: a fast spliced aligner with low memory requirements. Nat Methods. 2015;12(4):357–60. 25751142 10.1038/nmeth.3317 PMC4655817

[51] Li B, Dewey CN. RSEM: accurate transcript quantification from RNA-Seq data with or without a reference genome. BMC Bioinformatics. 2011;12:323. 21816040 10.1186/1471-2105-12-323 PMC3163565

[52] Sherman BT, Hao M, Qiu J, Jiao X, Baseler MW, Lane HC, et al. DAVID: a web server for functional enrichment analysis and functional annotation of gene lists. Nucleic Acids Res. 2022;50(W1):W216-21. 35325185 10.1093/nar/gkac194 PMC9252805

