# Supplementary figures and images for: Mining of candidate genes related to prolificacy in Jining grey goats using transcriptomics
Source: BMC Genomics. 2025 Dec 15;27:64. doi: 10.1186/s12864-025-12284-4 (PMC12821839; doi:10.1186/s12864-025-12284-4)

Supplementary Fig. 1 Results of the principal component analysis (PCA)

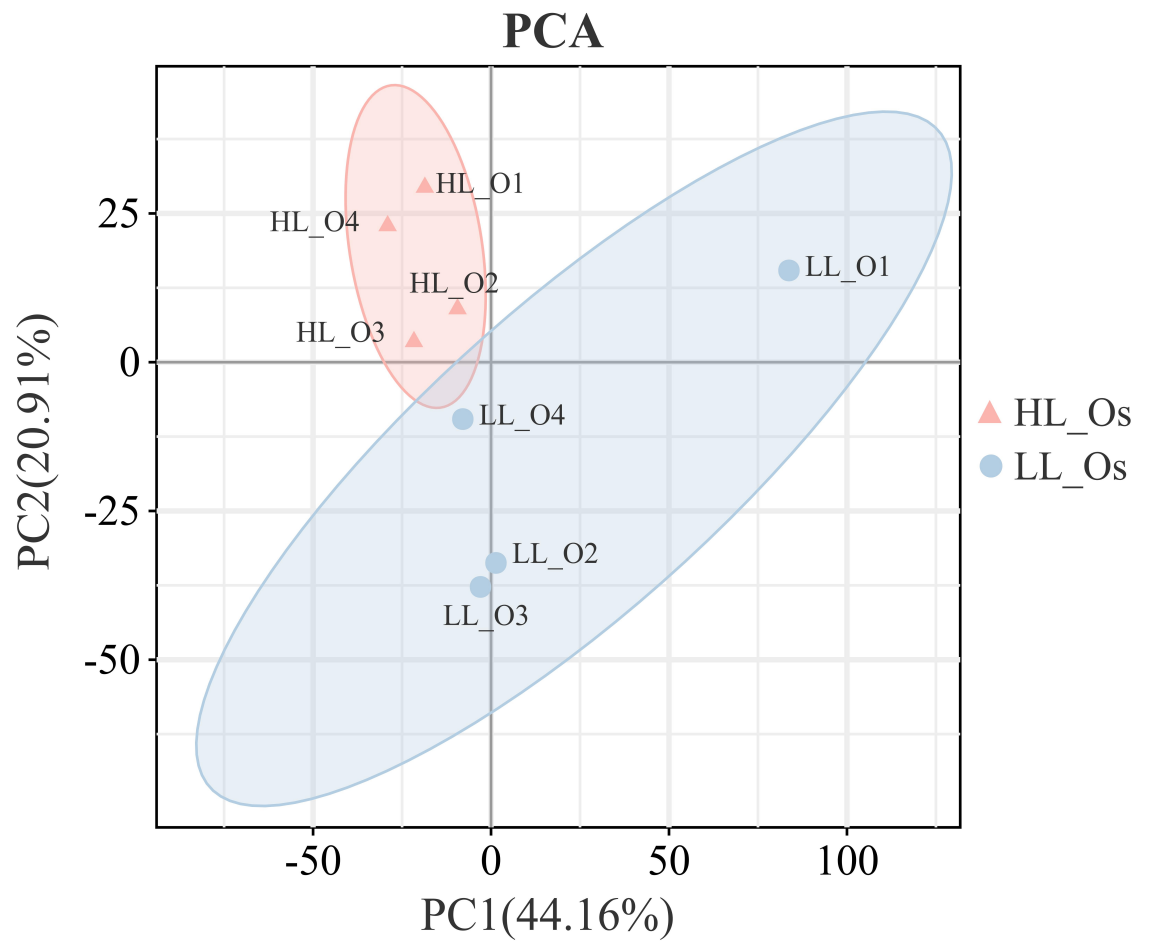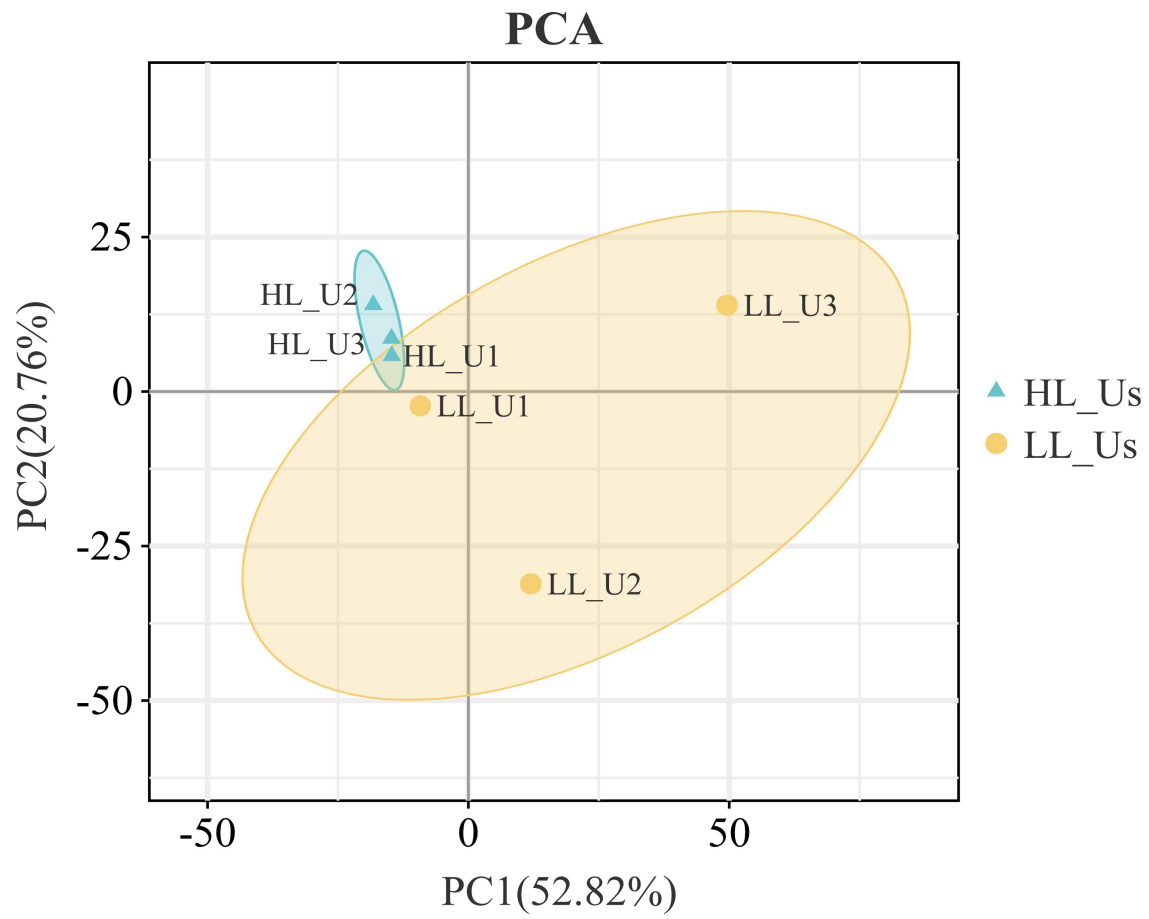

Supplement: Supplementary file 1 — Supplementary Material 1. [file 12864_2025_12284_MOESM1_ESM.pdf]
