# Supplementary material for: Mining of candidate genes related to prolificacy in Jining grey goats using transcriptomics
Source: BMC Genomics. 2025 Dec 15;27:64. doi: 10.1186/s12864-025-12284-4 (PMC12821839; doi:10.1186/s12864-025-12284-4)
Supplement: Supplementary file 2 — Supplementary Material 2. [file 12864_2025_12284_MOESM2_ESM.pdf]

Supplementary Fig. 2 Representative amplification and melting curves

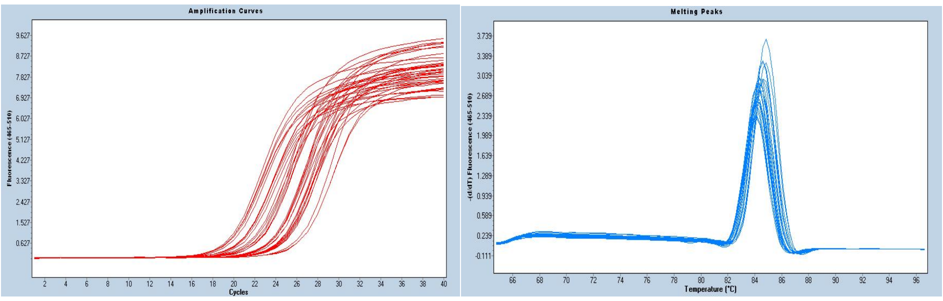

Ovary（3BHSD）

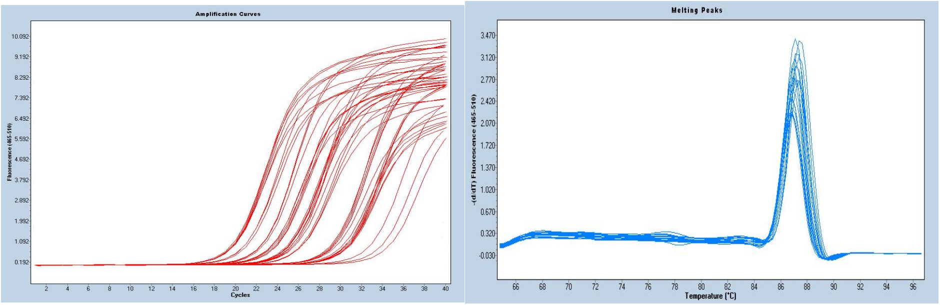

Ovary（CLSTN2）

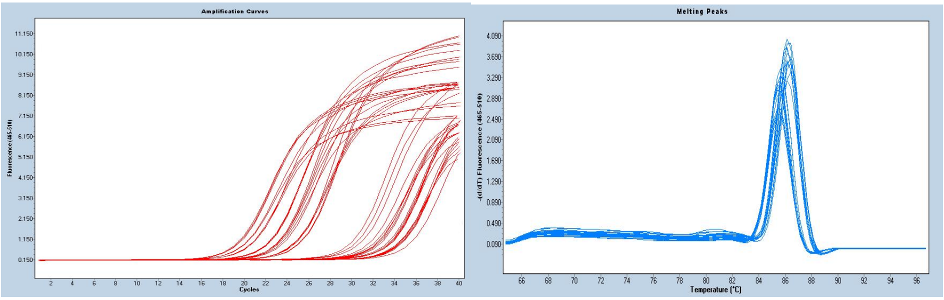

Ovary（BMP5）

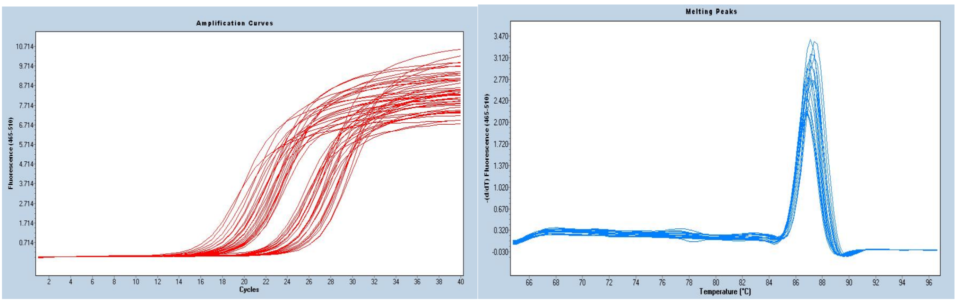

Ovary（DRD1）

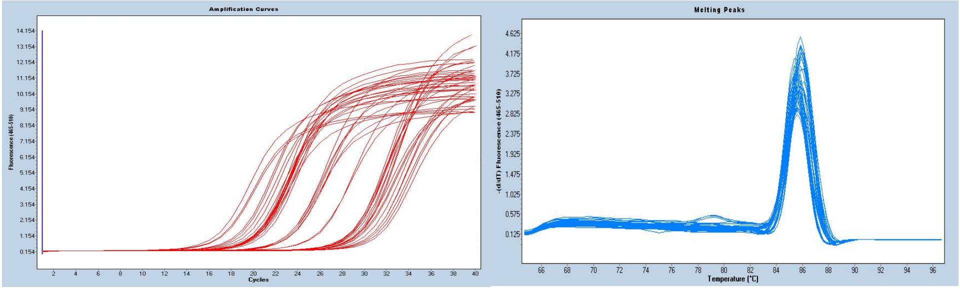

Ovary（ENO4）

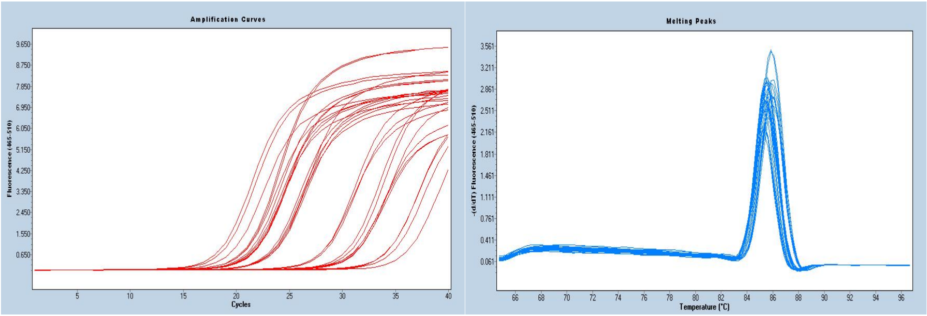

Uterus（DNAH1）

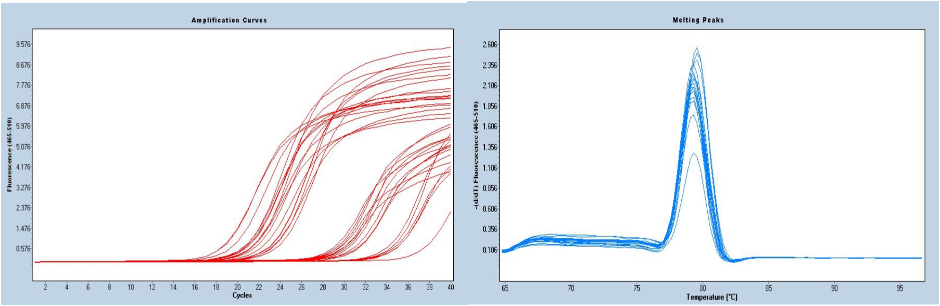

Uterus（GABRA1）

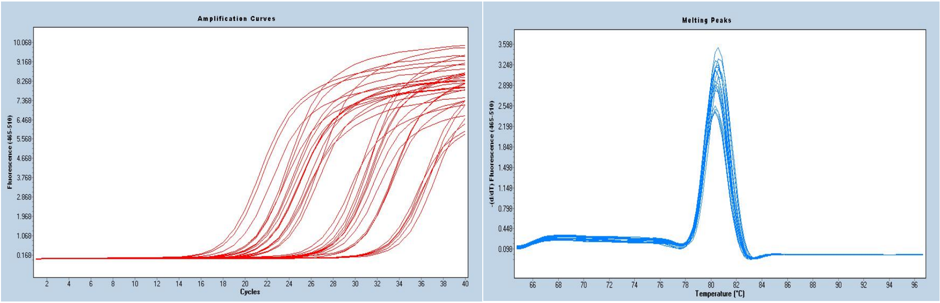

Uterus（CFAP43）

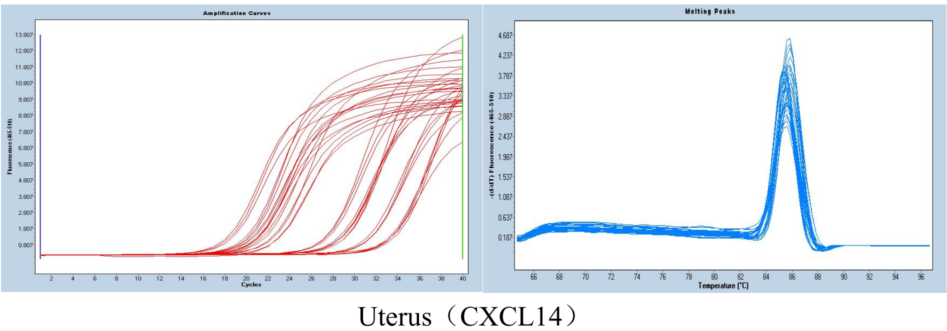

Uterus（CXCL14）

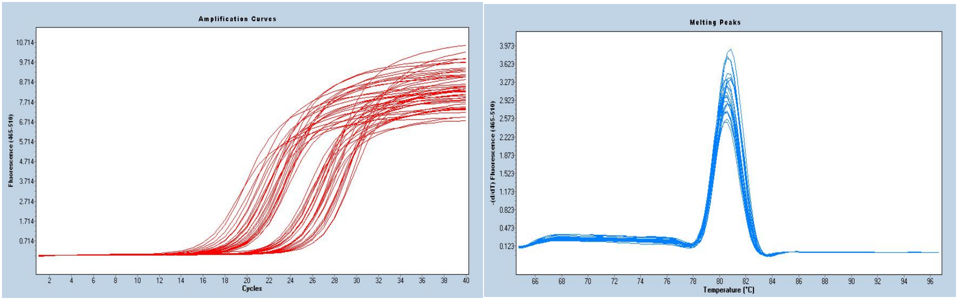

Uterus（WNT10B）
